# Experiments of the Effects of Alcohol on the Human Body

**Published:** 1871-08

**Authors:** E. A. Parkes, Count C. Wollowicz


*Experiments on the Effects of Alcohol on the Human Body.* By DR. E. A. PARKES and COUNT C. WOLLOWICZ.

An important series of experiments on this subject has been made conjointly by the authors. Their object was to ascertain the physiological and dietetic effects of alcohol on the human body in a state of health. The plan of observation was as follows:—For twenty-eight days, a man, an intelligent, healthy soldier, remained on a diet precisely similar as to food and times of meals in every respect, except that for the first eight days he took only water (in the shape of coffee, tea, and simple water); for the next six days he added to this diet rectified spirit, in such proportion that he took, in divided quantities, on the first day, one fluid ounce of absolute alcohol; on the second day, two fluid ounces; on the third day, four ounces; on the fifth and sixth days, eight ounces on each day. He then returned to water for six days, and then for three days took each day half a bottle (twelve ounces) of fine brandy, containing 48 per cent. of alcohol. Then for three days he returned to water. There were thus five periods, viz., of water drinking, alcohol, water, brandy, water. And for ten days before the experiments were commenced, the man, a beer-drinker usually, abstained from any alcoholic liquid. The food taken was all weighed; it was the ordinary diet. The general results obtained may now be very briefly summed up. It would seem, first of all, that, other conditions remaining the same, the weight of the body is unaffected by the taking of alcohol. With regard to temperature, we are told "that the general result from all observations surprised us (the observers), considering the numerous experiments on men and animals in which the temperature has been found to be lowered by alcohol." The tendency, indeed, was rather in the opposite direction, especially with the brandy. The alcohol was, however, never pushed far, because the object was not to induce any narcotism, but to ascertain its dietetic value; and the discrepancy involved in the observations of Drs. Parkes and Wollowicz may be in part further explained by the fact that the individual experimented upon possessed a perfectly healthy resisting, and not a diseased or weakened organism. The diminution of temperature by large and narcotic doses is not disputed; all that our experimenters affirm is, that with a small amount of alcohol and a good supply of food, the temperature is not diminished. The effects on the circulation described are very interesting. The pulse was not only more frequent and fuller when alcohol and brandy were used, but the increased frequency was persistent after the omission of the alcohol. The pulse had not reached in six days the point which was proper to it before the alcohol was given.

The first day of alcohol gave an excess of four per cent., the last twenty-three per cent., in the beats of the heart — that is, an excess of thirteen per cent. as a mean of six days. This, on calculation, amounts to an excess in the daily work of the heart equal to lifting 15.8 tons one foot during the first two, and 24 tons during the last two days of the alcoholic period. On the fifth and sixth days after the alcohol was left off, when its elimination was complete, the heart showed in the sphygmographic tracings signs of unusual feebleness; and when subsequently brandy was given, it was clear that it was acting upon a heart whose nutrition had not been perfectly restored. The observers say that it is evident that in the man experimented upon, the amount of alcohol the heart will bear without losing its healthy sphygmographic tracing is small, and it must be supposed that eventually some disease of the heart will follow the excitement induced by large doses of alcohol. The action on the kidneys of a moderate amount of alcohol is not marked; the amount of water eliminated is rather increased; no change takes place as regards the nitrogen when the ingress of nitrogen is constant, — certainly it is not diminished in amount. This conclusion is antagonistic to the observations formerly made on the point, which indicated that nitrogen is retained in considerable amount in the body under the exhibition of alcohol, which in this way increases assimilation, and when food is deficient saves the tissues from waste.

Little change is also effected in the phosphoric acid, the chlorine, and the free acidity of the urine. The elimination of nitrogen by the bowels was not lessened. The elimination of alcohol by the lungs was marked; indeed, a good deal must have been got rid of in this way,—by the skin considerable, by the kidneys slight. Drs. Parkes and Wollowicz think that, though not excessive at any one time, the exit is longer continued than Anstie and Dupre suppose.

Special note was taken of the effect of alcohol on digestion and appetite. It seems that in the man under observation, some point near two fluid ounces of absolute alcohol is the limit of useful action on appetite. It might have been found to be less had the experiments been continued. Further, although large doses interfered with the appetite, they did not interfere with or retard primary digestion, as far as could be seen, nor the normal chemical changes that result in the elimination of nitrogenous excreta, phosphoric acid, and the like. In a word, no evidence was forthcoming to show that alcohol either saved or exhausted the tissues; that is to say, the good or evil ascribed to alcohol in this direction was not observed by Drs. Parkes and Wollowicz in the healthy man. It may be, of course, different in disease. The effect on the nervous system was shown only by subjective symptoms —headache, heaviness, loss of cheerfulness and alacrity, torpor and sleepiness; and narcotism was induced by an amount of alcohol less than four and nearer two ounces daily, and the experimenters conclude that the narcotism, the loss of appetite, and the increased frequency in the heart's beats, are related to the common cause, viz., implication of the nervous system. The general inference of the experimenters on this point is, that something under two fluid ounces of alcohol could be taken daily without harm by the man under observation. The following are the final conclusions given by Dr. Parkes and his coadjutor. "It will be seen that the general result of our experiments is to confirm the opinion held by physicians as to what must be the indications of alcohol in health and disease. The effects on appetite and on circulation are the practical points to seize; and, if we are correct in our inferences, the commencement of narcotism marks the point when both appetite and circulation will be damaged. As to the metamorphosis of nitrogenous tissue, it seems improbable that alcohol in quantities that can be properly used in diet, has any effect; it appears to us unlikely (in the face of the chemical results) that it can enable the body to perform more work on less food, though by quickening a failing heart it may enable work to be done which otherwise could not be so. It may then act like the spur in the hide of the horse, eliciting force, though not supplying it." The experimenters, while recognizing further the great practical use of alcohol in raising a failing appetite, exciting a fainting heart, and accelerating a languid capillary circulation, are strongly impressed with the need of moderation and caution in its use. They do not deal with diseased conditions, but only a state of health, and do not refer at all to the action of wine or beer.—*Druggists' Circular and Chem. Gazette, Nov.,* 1870, *from London Pharm. Jour.*

